# How “Omics” Studies Contribute to a Better Understanding of Fuchs’ Endothelial Corneal Dystrophy

**DOI:** 10.3390/cimb47030135

**Published:** 2025-02-20

**Authors:** Erika Prašnikar, Spela Stunf Pukl

**Affiliations:** 1 Eye Hospital, University Medical Centre Ljubljana, 1000 Ljubljana, Slovenia; erika.prasnikar@kclj.si; 2 Faculty of Medicine, University of Ljubljana, 1000 Ljubljana, Slovenia

**Keywords:** Fuchs’ endothelial corneal dystrophy, FECD, omics study, genomics, transcriptomics, epigenomics, proteomics, metabolomics

## Abstract

Fuchs’ endothelial corneal dystrophy (FECD) is a progressive eye disease characterized by accelerated loss of endothelial cells and the development of focal excrescence (guttae) on Descemet’s membrane, resulting in cornea opacity and vision deterioration. The development of FECD is assumed to be due to the interplay between genetic and environmental factor risks, causing abnormal extracellular-matrix organization, increased oxidative stress, apoptosis and unfolded protein response. However, the molecular knowledge of FECD is limited. The development of genome-wide platforms and bioinformatics approaches has enabled us to identify numerous genetic loci that are associated with FECD. In this review, we gathered genome-wide studies (n = 31) and sorted them according to genomics (n = 9), epigenomics (n = 3), transcriptomics (n = 15), proteomics (n = 3) and metabolomics (n = 1) levels to characterize progress in understanding FECD. We also extracted validated differentially expressed/spliced genes and proteins identified through comparisons of FECD case and control groups. In addition, highlighted loci from each omics layer were combined according to a comparison with similar study groups from original studies for downstream gene-set enrichment analysis, which provided the most significant biological pathways related to extracellular-matrix organization. In the future, multiomics study approaches are needed to increase the sample size and statistical power to identify strong candidate genes for functional studies on animal models and cell lines for better understanding FECD.

## 1. Introduction

The human cornea is the outermost portion of the eye, which from the anterior to the posterior surface consists of the epithelium, the Bowman’s layer, the stroma, the Descemet’s membrane, and the endothelium. The endothelium is a monolayer of hexagonal cells, which regulates the hydration of the corneal stroma and maintains the transparency of the cornea by the “pump-leak” system. Their intercellular tight junctions act as a porous barrier which allows aqueous humor to passively diffuse through the barrier into the corneal stroma to deliver oxygen and nutrients to the tissue, and they also actively transport excess fluid out of the stroma via a sodium (Na^+^) potassium (K^+^) pump to keep the cornea dehydrated [1]. Endothelial cells in adult human cornea have no mitotic activity in vivo, meaning they do not proliferate [2]. Scientists are now investigating whether these cells still have some mitotic activity, particularly the cells in the corneal periphery. The migration of these cells may be augmented by pharmacological agents such as Rho kinase inhibitors [3]. However, in natural conditions, the density of endothelial cells over a lifetime gradually decline at an average rate of 0.6% per year [4]. It was determined that, in second decade of life, the density is approximately 3400 endothelial cells/mm^2^, with 2600 cells/mm^2^ in the fifth and 2300 cells/mm^2^ in the ninth decade. The space once occupied by now-dead endothelial cells is taken up by the enlargement and migration of the surrounding cells, which consequently leads to an increased rate of variations in size (polymegethism) and shape (pleomorphism) [5]. Endothelial counts below 500 cells/mm^2^ may lead to the development of corneal edema, which impacts transparency and results in visual disturbance [2,6].

**Figure 1:**
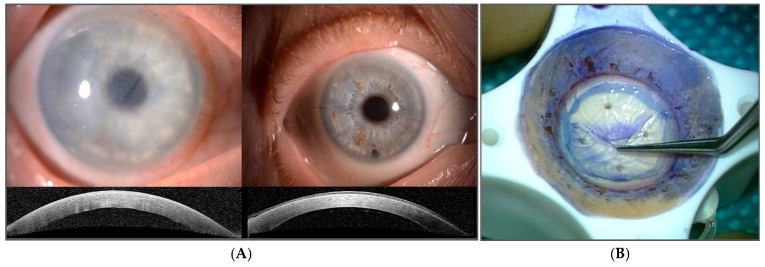
(**A**) Biomicroscopic photography (above) and optical coherence tomography (below) of the cornea of a patient with Fuchs’ endothelial corneal dystrophy with stromal edema and opacity (left) and after normal postoperative status with clear corneal and normal stromal transparency (right) after endothelial corneal transplantation. (**B**) Intraoperative view of corneal tissue during Descemet membrane stripping for endothelial corneal transplantation.

Fuchs’ endothelial corneal dystrophy (FECD) is a corneal pathology that is characterized by a more rapid decline of endothelial cells’ density and the formation of extracellular-matrix (ECM) excrescences in the Descemet’s membrane, termed guttae. The progressive loss of endothelial cell disturbs the pump–leak mechanism of the endothelium, causing fluid to accumulate in the stroma. As the result, the cornea becomes edematous, the vision disturbed and blurry, and if not treated it can lead to corneal blindness (Figure 1A). FECD is currently the leading indication for the cornel transplantation [7] (Figure 1A,B). There are genetic predispositions, which are further discussed below, associated with FECD. However, the disease’s development and progression is thought to be multifactorial, also depending on environmental factor risks, such as ultraviolet A (UVA) light exposure, estrogen metabolism, obesity, and smoking [8,9,10,11].

The pathogenesis of FECD includes an increased production of reactive oxygen species (ROS) in the corneal endothelium, causing the accumulation of nuclear and mitochondrial endothelial DNA damage. This further contributes to endothelial cell apoptosis, senescence, mitochondrial burnout, abnormal ECM in the form of guttae, impairment in the endothelial-to-mesenchymal transition (EMT) for the renewal and repair of endothelial gaps, and unfolded protein response due to an accumulation of misfolded proteins in the endoplasmic reticulum [12,13,14,15,16,17].

Clinically, FECD is classified into two forms, early-onset and late-onset FECD. The early-onset FECD is rare and manifests early in life, becoming symptomatic in the second or third decade of life. The more common late-onset FECD begins in the fifth or sixth decade of life and progresses thereafter through four clinically defined stages [17]. The prevalence of late-onset FECD differences among ethnic populations, from 3.7% in Japanese, 6.7% in Chinese Singaporeans [18], 9% in Icelanders [19], to 11% in the Tangier islanders [20], and is more common in women.

The term “omics” refers to a special group of experiments with microarrays, next-generation sequencing (NGS), or mass spectrometry platforms, which enable a comprehensive or genome-wide assessment of biological samples. The platforms generate high-throughput data for downstream analyses, using different computational approaches to identify differences between study and control groups [21].

Five main omics levels exist: genomics, epigenomics, transcriptomics, proteomics, and metabolomics, depending on the analyzed biologic material. Genomics studies are performed at the DNA level and provide a framework for mapping and studying specific genetic variants contributing to mendelian or complex diseases/traits. The variants could be single nucleotide polymorphisms (SNPs), nucleotide insertion or deletion (indels) causing start/stop-loss or -gain variants, non-synonymous or non-frameshift substitution, or non-frameshift or frameshift insertion/deletion to the RNA transcripts that further affect the amino-acid sequence or splice site variants [21]. The whole-genome sequencing (WGS) approach enables analyzing the entire genome, including protein-coding (exome) and non-coding (intron) regions, but the bioinformatics identification of candidate genetic variants is time consuming and complex. Therefore, NGS technology enables the sequencing of the region of interest, consequently reducing the amount of sequences that need to be generated and analyzed by bioinformatics approaches [22]. As it is estimated that about 85% of mutations with major effects on disease-related traits are located in the exome, the whole-exome sequencing (WES) approach is often applied, as it requires only about 2% of the sequencing “load” compared to WGS. Epigenomics studies are orientated to identify a reversible modification of DNA (DNA methylation patterns) or DNA-associated proteins (histone acetylation) which are tissue-specific and major regulators of gene transcription. Transcriptomics studies are focused on the global characterization of coding and non-coding RNA transcripts in examined tissue/cell samples. Bioinformatics RNA-sequencing (RNA-seq) data analysis by read alignment to the reference genome enables the identification and quantification of expressed genes and the determination of alternative splicing variants [23]. Studies of proteomics levels are focused on the quantification of peptide abundance, modification, and interaction. Metabolomics studies quantify small molecules of cellular metabolism, such as amino acids, fatty acids, and carbohydrates. Intergenomics approaches search for possible overlaps between the results of different omics layers [21,24].

Identified differentially methylated/expressed/spliced loci between study groups can be further analyzed by bioinformatics tools to reveal their biological meaning through key biological pathways and genes. The gene-set enrichment analysis (GSEA) method is designed to group genes with a common biological function or regulation into enriched pathways and processes on the basis of prior biological knowledge, thus providing an insight into the biological mechanisms of an investigated disease/trait [25].

There is an accumulation of omics-based studies in the filed of ophthalmology aiming to identify the reliable biomarkers in different eye diseases, such as age-related macular degeneration, diabetic retinopathy, retinal detachment, myopia, glaucoma, cataract, keratoconus, dry eyes, and FECD [26]. During our literature search, we found review articles summarizing the genetic loci associated with FECD based on candidate gene studies [27,28,29], but no review article summarizing omics studies has focused on FECD patients. The aims of this review article were: 1. to perform a literature search for the omics studies performed on FECD; 2. to sort the retrieved studies according to the omics levels; 3. to extract methodological information and the main outcomes of the studies; 4. to perform data synthesis of the loci associated with FECD from retrieved studies in order to perform a GSEA.

## 2. Literature Selection and Study Overview

**Figure 2:**
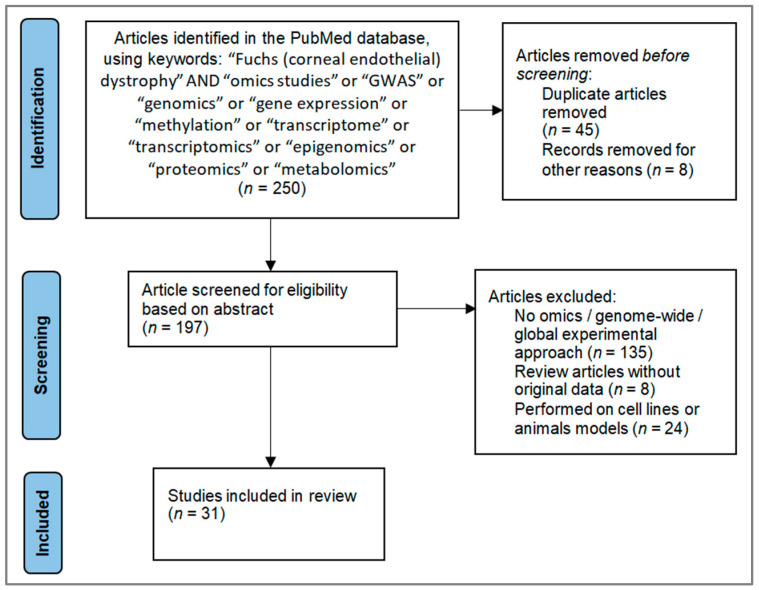
A flow diagram summarizing the literature screening method and study selection process. Adapted from the Preferred Reporting Items for Systematic Reviews and Meta-Analyses (PRISMA).

**Figure 3:**
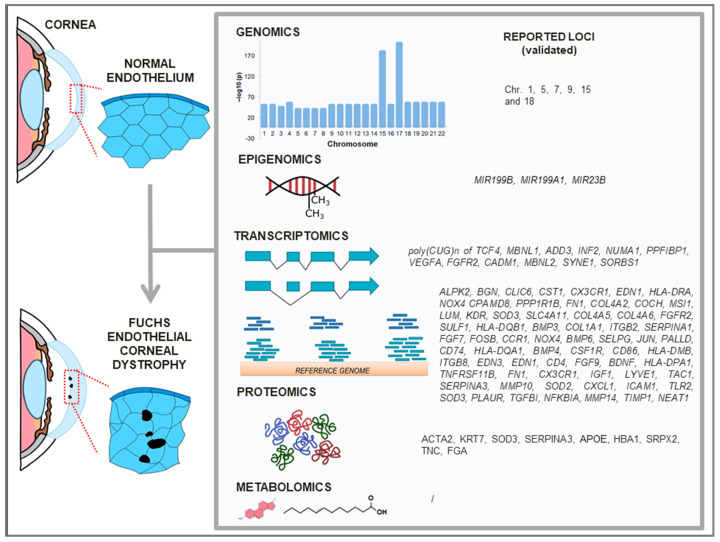
Genetic loci associated with dysregulation in corneal endothelium of FECD that were identified by genome-wide studies. Retrieved studies were sorted according to genomics, epigenomics, transcriptomics, proteomics, or metabolomics level and only loci that were further confirmed by validation analyses were extracted.

The literature search (Figure 2) for articles published up to December 2024 was performed in the PubMed database using “Fuchs (corneal endothelial) dystrophy” AND “omics studies” or “GWAS” or “genomics” or “gene expression” or “methylation” or “transcriptome” or “transcriptomics” or “epigenomics” or “proteomics” or “metabolomics” as search tags. After the retrieval of 250 articles, each article was manually reviewed to select relevant studies that met specific criteria, including the omics/genome-wide/global approach used on blood, corneal endothelium, and aqueous humor or Descemet’s membrane samples from patients with FECD, as well as excluding studies involving animal samples or (immortalized) corneal endothelial cell lines. At the end, 31 articles were included and further sorted according to study design into genomics (9 articles), epigenomics (3 articles), transcriptomics (15 articles), proteomics (3 articles), or metabolomics (1 article). A graphical abstract of the review is presented in Figure 3. At each omics level, validated loci identified as associated with FECD were extracted from the studies.

## 3. Genomics of FECD

During the literature search, we retrieved nine genome-wide studies related to FECD that were performed at the genomics level (Table 1). Four of them analyzed the general population, four analyzed family members, and one study analyzed both groups of FECD patients.

### 3.1. Family-Based Studies

FECD is suggested to be inherited in an autosomal dominant pattern, as the disease is a common feature in members of affected families. The first microsatellite-based genetic linkage analyses of families with FECD reported candidate chromosomal loci 1p34.3-p32 [30], 13pTelomere-13q12.13 [31], and 18q21.2-q21.3 [32].

Riazuddin et al. [33] applied SNP microarray genotyping, where SNP markers rs13173656, rs9313417, rs2116736, rs17451810, rs4958561, and rs778816 identified a novel locus at 5q33.1-q35.2 as the most associated with FECD. A genome-wide linkage scan of 22 families with FECD performed by Afshari et al. [34] reported additional five regions on chromosomes 1 (rs760594), 7 (rs257376), 15 (rs352476), 17 (rs938350), and X (rs548996). In a following study, Riazuddin et al. [35] applied NGS to 12 individuals affected by late-onset FECD from a three-generations family and found an association with a nonsense mutation in *AGBL1* at the 15q locus. In attempts to identify additional candidate genes for FECD, Jiang et al. [36] conducted a WES approach and downstream linkage analysis on 8 members from a five-generation Chinese family affected by FECD with or without anterior polar cataract. They managed to identify two candidate genes: *INTS1* located on chromosome 7 and *SH3GL2* located on chromosome 9. However, they could not achieve genotype–phenotype co-segregation in a further validation analysis in the whole 33 family members [36].

### 3.2. Population-Based Studies

It is believed that the heritability in heterogeneous diseases is attributed to variants (SNPs) with a minor allele frequency above 1% in the general population, with each variant having a small additive effect on the disease phenotype [37].

To understand the genetic etiology of FECD, Baratz et al. [38] enrolled 280 FECD case and 410 control subjects of European ancestry and performed a genome-wide association study (GWAS). They identified SNP rs613872 in the locus of transcription factor 4 (*TCF4*), which is located at 18q21.2, to be associated with the risk of FECD. Similar results were reported by Li et al. [39]. Afshari et al. [40] also applied GWAS on 1404 FECD cases and 2564 controls of European ancestry and reported additional three novel loci *KANK4* (rs79742895), *LAMC1* (rs3768617), and *LINC00970*/*ATP1B1* (rs1200114) [40].

Several groups have confirmed genetic variations (SNPs, mutations, and trinucleotide repeat (TNR) expansion) in candidate genes, including *COL8A2*, *TCF4*, *SLC4A11*, *AGBL1*, *ZEB1*, *LOXHD1*, *XRCC1*, *FAS*, and *FASLG*, to be in an association with early-onset and/or late-onset FECD [30,39,41,42,43,44,45,46,47,48]. The roles of the most common genes associated with the pathophysiology of FECD have been reviewed elsewhere [17,27]. However, not all studies confirmed a positive association between candidate genes and FECD, and some studies confirmed the positive association only in a small proportion of cases [49,50,51,52,53,54]. In addition, the associations between the candidates’ risk alleles and FECD differ according to the ethnic background of the patients. For example, the association between FECD and intronic rs613872 of the *TCF4* gene, reported in both GWAS studies [38,40], was confirmed in FECD patients from Germany [55], Greece [56], Midwestern United States [57], and Australia [58], but not in patients from China [59] and Thailand [60]. However, Wieben et al. [61] identified an intronic TNR cytosine–thymine–guanine (CTG) expansion (>50 repeats) within *TCF4* with a better predictive value for FECD compared to the rs613872 of *TCF4.* The association between intronic CTG TNR expansion (also termed *CTG18.1* in some publications) in *TCF4* and FECD has been confirmed by other groups [44,62].

In 2023, Peachey et al. [63] performed a multi-ancestry GWAS, including participants of European, African, and Hispanic/Latino ancestries, and identified a significant association between the rs11659764 of *TCF4* and FECD. When performing a meta-analysis between European and African datasets, they reported eight significant novel loci; *LAMA5*, *LAMB1*, *COL18A1*, *SSBP3*, *THSD7A*, *RORA*, *PIDD1*, and *HS3ST3B1*.

### 3.3. Mitochondrial DNA (mtDNA)

Czarny et al. [64] demonstrated extended mtDNA damage and a higher rate of the common 4977 bp mtDNA deletion in corneal endothelial cells compared to matched peripheral blood lymphocytes isolated from FECD patients. They suggested that the oxidative stress is a major player in the pathogenesis of FECD and that the corneal endothelial mitochondria are its main target [64]. A circular mtDNA encodes 37 mitochondrial genes and it is transmitted maternally without undergoing recombination. Therefore, it has been widely used to understand human evolutionary patterns across continents. Mitochondrial DNA haplogroups were established according to 10 SNPs, which differ depending on ethnic group, and some have been already associated with different eye diseases, including keratoconus [65], age-related macular degeneration [66], and primary open-angle glaucoma [67].

Li et al. [8] used published datasets of genotyped 4228 individuals of European ancestry to investigate whether mtDNA variants are associated with FECD. They reported that the minor 10398G allele (rs2853826) in the NADH dehydrogenase 3 (*ND3*) gene and mitochondrial Haplogroup I decrease the risk for FECD. According to the findings, it has been suggested that mtDNA may be a specific target of increased oxidative stress in FECD due to some environmental risk factors, such as smoking [8].

**Table 1:** Extracted data from genomics studies performed on FECD. FECD cases in the discovery group refer to late-onset FECD patients, but if the clinical classification of the FECD cohort was not clearly defined in the origin study, the number of included FECD cases is written in *italic* font. Abbreviations: APC = anterior polar cataract. CE = corneal endothelium. Chr = chromosome. IHC = immunohistochemistry. PCR = polymerase chain reaction. SNP = single nucleotide polymorphism. LOD = logarithm of odds. HLOD = heterogeneity LOD. / indicates no data/information in the original study.

Ref.	Relation of Participants (Raw Data Accession)	Discovery Group		Replication Group		Genomic Approach	Genome-Wide Platform	Statistical Approach	Reported Loci (SNPs) Associated with FECD; Statistical Significance	Validation of Identified Loci
		FECD Cases	Controls	FECD Cases	Controls					
[8]	European population (datasets from [39] and dbGaP database: phs000421.v1.p1and phs000429.v1.p1)	*530 + 457*	498 + 498	*857 + 857*	2342 + 2186	Whole-genome	/	Logistic regression model	rs2853826 (the variant A10398G) of *ND3* (*p* = 0.034) and Haplogroup I (*p* = 0.041)	/
[33]	Three-generation family	11	6	/	/	Whole-genome	Affymetrx SNP microarry ver. 5.0	Parametric two-point linkage and haplotype analyses; autosomal dominant model	Chr5 (rs13173656, rs9313417, rs2116736, rs17451810, rs4958561, rs778816); LOD > 2	Confirmed by short-tandem-repeat microsatellite markers
[34]	Large multigenerational family and 21 small multiplex families	56	36	/	/	Whole-genome	Illumina SNP linkage panel IV	Parametric two-point and nonparametric multipoint linkage analyses; autosomal dominant and recessive models	Two-point analysis: Chr1 (rs760594), Chr7 (rs257376, rs1047035), Chr15 (rs352476, rs1075991), Chr17 (rs938350), ChrX (rs548996); HLOD > 1.5. Multipoint analysis: Chr7 (rs740295, rs918980), Chr17 (rs1530348); LOD > 1.5.	/
[35]	Three-generation family	12	3	/	/	Whole-exome	Illumina HiSeq2000 Genome Analyzer	In-house pipeline	Chr15 (nonsense mutation c.3082C > T in *AGBL1*)	Confirmed by dideoxy sequencing and low gene expression in CE by qPCR
[36]	Five-generation Chinese family	*9 + 5 (coexisted APC)*	19	/	/	Whole-exome	Illumina HiSeq X-Ten	Multipoint parametric and nonparametric linkage analyses; autosomal dominant models	Linkage analyses: no candidate region with LOD > 2. After additional data filtering: Chr7 (*INTS1*) and Chr9 (*SH3GL2*)	Confirmed by PCR and Sanger sequencing
[39]	Caucasian 64 multiplex families	165	50	/	/	Whole-genome	Illumina Golden- Gate linkage panel IVB and Infinium Human- Linkage12	Parametric and nonparametric two-point and multipoint analyses; dominant and recessive models	Multipoint dominant model: Chr18 (rs1145315); HLOD = 2.5. Two-point analysis: Dominant model: Chr10 (rs1889974), Chr15 (rs235512). Recessive model Chr19 (rs893186); HLOD > 3.	/
	Caucasian population	450	340	/	/	Whole-genome	/	Association analysis; dominant, additive and recessive models	Chr18 (rs613872 *TCF4*) for all three genetic models (*p* < 0.05; with *p* = 9.33 × 10^−35^ in dominant model)	Confirmed by PCR
[38]	European population (available at dbGaP: phs000246.v2.p1)	*130*	260	*150*	150	Whole-genome	Illumina 370K Beadchip	Log-additive model (SAS software, v.9.1.)	Chr 18 (rs613872 *TCF4*); *p* = 2.34 × 10^−26^	/
[40]	European population (available at dbGaP: phs000001.v3.p1)	*1404*	2564	*671*	778	Whole-genome	Illumina Omni2.5- 4v1_H array	Logistic regression with additive model	Chr1 (rs79742895 *KANK4*, rs3768617 *LAMC1*, rs1200114 *LINC00970/ATP1B1*), Chr18 (rs784257 *TCF4*); (*p* < 5 × 10^−8^)	Gene and protein expression in CE confirmed by RNA-seq and IHC, except *KANK4* only by IHC.
[63]	European, African, Hispanic/Latino populations (datasets also obtained from [40])	*2251*	252,345	*1404*	2564	Whole-genome	Thermo Fisher MVP 1.0 Axiom array	SAIGE v1.1.6.2, +mungle plug-in (bcftools v1.16)	Chr1 (rs79742895 *KANK4*, rs1200114 *ATP1B1*, rs2093985 *LAMC1*, rs11590557 *SSBP3*), Chr7 (rs74882680 *THSD7A*, rs150990106 *LAMB1*), Chr11 (rs1138714 *PIDD1*), Chr15 (rs12439253 *RORA*), Chr17 (rs9303111 *HS3ST3B1*), Chr18 (rs11659764 *TCF4*), Chr20 (rs141208202 *LAMA5*), Chr21 (rs114065856 *COL18A1*); *p* < 10^−8^	/

## 4. Transcriptomics of FECD

Several studies examined the expression patterns of candidate genes in the corneal endothelium of FECD patients [27,68]. Eghrari et al. [69] identified 11 different isoforms of *TCF4* expressed in healthy corneal endothelium, including a novel isoform called 7b [69]. We have gathered the transcriptomics studies performed on corneal endothelium samples of patients with FECD in Table 2 and summarized them below.

According to some transcriptomics studies, corneal endothelial gene-expression patterns differ among FECD patients depending on the presence or absence of a pathogenic intronic CTG TNR expansion (hereafter referred to as intronic TNR expansion) in the *TCF4* gene. A distinct study-group clustering was observed by Bhattacharyya et al. [70] when they performed a principal component analysis (PCA) of RNA-seq data obtained from primary corneal endothelial cell cultures of FECD patients with and without intronic TNR expansion within *TCF4* [70]. On the other hand, Zhang et al. [71] observed similar global transcriptome signatures among FECD cohorts when performing a transcriptomics meta analysis [71].

### 4.1. Alternative Splicing Events

Du et al. [72] performed RNA-seq of the corneal endothelial tissue samples of five FECD patients and four normal controls to examine the gene-expression patterns of *TCF4* with intronic TNR expansion and to identify additional alternative splice isoforms. They detected intronic TNR expansion-containing TCF4 transcripts (hereafter referred to as poly(CUG)*n* TCF4 transcripts) in three FECD patients with a confirmed intronic TNR expansion in the *TCF4* gene, while expression of normal TCF4 transcripts was found in one FECD patient and all controls without intronic TNR expansion in *TCF4*. Further analysis of RNA-seq data revealed additional mRNA splicing changes in 342 genes, including *MBNL1*, *INF2*, *ITGA6*, *ADD3*, and *SORBS1*, in FECD cases with intronic TNR expansion in *TCF4*. When applying RNA fluorescence in situ hybridization (RNA-FISH) on the fibroblast and corneal endothelium of FECD cases and controls, they detected RNA foci containing misspliced poly(CUG)*n* TCF4 transcripts and the associated protein muscleblind-like splicing regulator 1 (MBNL1) in the corneal endothelium of the FECD cohort with an intronic TNR expansion in *TCF4*. They concluded that poly(CUG)*n* TCF4 transcripts predominantly accumulate in the corneal endothelium, contributing to FECD pathogenesis [72].

Wieben et al. [73] performed RNA-seq of corneal endothelium samples from 11 FECD patients and 4 healthy controls with and without intronic TNR expansion in *TCF4*, respectively. They identified 61 alternatively splicing events in 58 genes and exposed splicing changes within *NUMA1*, *PPFIBP1*, *MBNL1*, and *MBNL2* transcripts as the most representative for the FECD disease. An enrichment analysis of differentially spliced genes using Panther bioinformatics tool revealed molecular functions involved in cytoskeletal protein binding and cell adhesion. Similar as Du et al. [72], they concluded that missplicing events in FECD corneal endothelium cause RNA toxicity, characterized by the binding of poly(CUG)*n* TCF4 transcripts with the splicing factors MBN1 and MNB2 and further accumulation in RNA foci [73]. RNA toxicity due to the dysregulation of TCF4 isoform was also demonstrated by spatial transcriptomics analysis on primary corneal endothelial cell culture from FECD patients with intronic TNR expansion in *TCF4* [70].

In a further RNA-seq study [74], Wieben et al. added a FECD cohort (n = 6) without intronic TNR expansion within *TCF4* to check for differences in gene expression and RNA splicing patterns between the FECD cohort with intronic TNR expansion in *TCF4*. They identified 20 differentially splicing events in *MBNL1*, *NUMA1*, *APBB2*, *PPFIBP1*, *INF2*, *SCARB1*, *SYNE1*, *ADD3*, *MBNL2*, *TTC7A*, *ARVCF*, *TSPOAP1*, *NDUFV3*, *IFI44*, *EXOC1*, *ITGA6*, *CLASP1*, *COPZ2*, *CD46*, and *CADM1*. In FECD patients with intronic TNR expansion within *TCF4*, the inclusion of an additional exon in the ADD3 and CADM1 transcripts and the exclusion of an exon sequence in the INF2 transcript were confirmed by the reverse-transcription PCR (RT-PCR) technique. Further gene-expression analysis revealed 28 upregulated and 11 downregulated genes in the FECD cohort with an intronic TNR expansion in *TCF4* which provided no significant enriched pathways. In addition, they used RNA-seq data to check for any mutation in genes *COL8A2*, *SLC4A11*, *TCF4*, *ZEB1*, and *DMPK* which are generally associated with FECD, but they have not found any rare variants. Furthermore, they have not observed any expression levels of *LOXHD1* and *AGBL1*, which are also generally associated with FECD, in their tissue samples. However, in one sample from the FECD cohort without an intronic TNR expansion in *TCF4*, they identified a rare genetic variant named hg19 chr17:g.56383714 C > T, resulting in the substitution of an arginine to a histidine at amino acid 1738 (R1738H) in TSPOAP1. A significant amount of misspliced TSPOAP1 transcripts in the FECD cohort with intronic TNR expansion in *TCF4* were associated with the deletion of a 180 amino-acid-long part of a TSPOAP1 motive that interacts with TSPO, a mitochondrial outer-membrane protein involved in the transport of cholesterol in the mitochondria. It is thought that TSPO is an important regulator of mitophagy, the dysregulation of which is involved in FECD pathogenesis. A rare variant of *TSPOAP1* identified in a FECD participant lacking the intronic TNR expansion in *TCF4* was confirmed on her leukocyte DNA by Sanger sequencing. Further exon-sequencing analysis of her family revealed rare TSPOAP1 variants in two affected members, indicating the possibilities of some common pathological mechanisms with FECD patients with intronic TNR expansion in *TCF4* [74].

Chu et al. [75] conducted RNA-seq of corneal endothelium samples from four study groups, which were also designed according to the results of *TCF4* genotyping. They included FECD patients with intronic TNR expansion in *TCF4* (n = 6), FECD patients without intronic TNR expansion in *TCF4* (n = 4), pre-symptomatic eye donors with intronic TNR expansion in *TCF4* (n = 6) and normal controls without intronic TNR expansion in *TCF4* (n = 9). RNA-seq data analysis revealed a clustering of samples into two groups; control and pre-symptomatic in one group, and both FECD subgroups in the other group. It was shown that the rate of splicing changes, most commonly skipped exons, was higher in all three study groups compared to the control group. Splicing changes were also observed in the splicing factors *MBNL1* and *MBNL2*, further hypothesized to trigger the accumulation of splicing changes leading to late-stage FECD. Identified splicing changes in six genes, *INF2*, *NUMA1*, *SORBS1*, *SYNE1*, *MBNLL1* and *MBN2*, were confirmed by RT-PCR.

**Table 2:** Extracted data from transcriptomics studies performed on corneal endothelium in FECD patients. F_RE+ refers to FECD patients with genotyped intronic trinucleotide repeat (TNR) expansion within *TCF4*. F_RE− refers to FECD patients with confirmed absence of intronic TNR expansion within *TCF4*. n.s. refers to no data regarding the genotyping of intronic TNR expansion in *TCF4*. CO_RE+ refers to FECD pre-symptomatic donated corneas with confirmed intronic TNR expansion in *TCF4*. CO refers to control corneas without intronic TNR expansion within *TCF4*, or information not reported in the study. ↑ and ↓ refer to up- and downregulated genes, respectively, in FECD compared to control groups. Abbreviations: DEGs = differentially expressed genes. DSGs = differentially spliced genes. ASEs = alternative splicing events. FDR = false discovery rate. qPCR and RT-PCR = quantitative and reverse-transcriptase polymerase chain reaction, respectively. IHC = immunohistochemistry. FISH = fluorescence in situ hybridization. FC = fold change. GEO = Gene-Expression Omnibus database. poly(CUG)*n* TCF4 = TCF4 transcripts containing an intronic trinucleotide repeat expansion. / indicates no data/information in the original study.

Ref.	Available Raw Data: Accession Number	Study Design	FECD Cases (Ancestry, Average Age)			Controls (Ancestry, Average Age)		Genome-Wide Platform	Bioinfor-Matics Tool/Software	Significant Loci Associated with FECD Cohort Relative to CO Comparison		Validation of Identified Locus/Loci
			F_RE+	F_RE−	n.s.	CO_RE+	CO			Differentially Expressed Genes (DEGs)	Differentially Spliced Genes (DSGs) or/and Alternative Splicing Events (ASEs)	
[71]	Extracted from GEO: GSE142538, GSE112201; SRA: PRJNA524323	Meta analysis	25	8	/	/	19	/	DEGs: AWFisher DSGs: rMATS turbo	1184 ↑, 1018 ↓; FDR < 0.05	DSGs: No data provided. Prevalence of exon skipping events among datasets; FDR < 0.05	/
[72]	/	Differ. splicing analysis	4	/	/	/	3	Illumina HiSeq 2000	Limma CASPER v1.9.0	/	342 DSGs; *p* < 0. 05. Identified poly(CUG)*n* TCF4 transcripts	Confirmed poly(CUG)*n* TCF4 by FISH. Confirmed ASE of MBNL1 (inclusion of exon 6), ADD3 (inclusion of exon 14) and INF2 (exclusion of exon 22) by RT-PCR
[73]	/	Differ. splicing analysis	11 (72 years)		/	/	4 (63 years)	Illumina HiSeq4000	Mayo Analysis Pipeline for RNA-seq	/	58 DSGs/61 ASE	Confirmed ASE for NUMA1 (exon exclusion), PPFIBP1 (exon exclusion), VEGFA (exon inclusion), FGFR2 (exon exclusion) by RT-PCR and MBNL2 (exon inclusion) by RT-PCR and Sanger sequencing
[74]	Deposited to GEO: GSE112201	Differ. splicing and gene expression analyses	18 (71 years)	6 (68 years)	/	/	/	Illumina HiSeq2000 or HiSeq4000	DSGs: MISO DEGs: edgeR and z-test	28 ↑, 11 ↓; log2 FC > 1	20 ASE	Confirmed ASE of ADD3 (exon inclusion), CADM1 (exon inclusion), INF2 (exon exclusion) by RT-PCR in F_RE+ group
[76]	Deposited to GEO: GSE171830	Differ. gene expression analysis	/	/	9 (pooled)	/	3	Illumina HumanHT-12 v4.0	Limma	126 ↑, 16 ↓; Log2 FC ≥ 1.5, FDR ≤ 0.05	/	Confirmed ↑ of *ALPK2*, *BGN*, *CLIC6*, *CST1*, *CX3CR1*, *EDN1*, *HLA-DRA*, *NOX4* and ↓ of *CPAMD8* and *PPP1R1B* by qPCR
[77]	Extracted from GEO: GSE74123, GSE171830, GSE101872, GSE142538; SRA: PRJNA524323	Meta analysis	19 (Cauc-asians)	9 (Cauc-asians)	13 (Caucasia-ns)	/	26 (Cauc-asians)	/	Combined effect size method. Random effect model.	1103 ↑, 434 ↓; FDR < 0.05	/	/
[78]	Deposited to DNA Data Bank of Japan: DRA015078 and Genomic Expression Archive: E-GEAD-564	Differ. gene expression analysis	/	/	10 (late-onsetCaucasia-ns, 67 years)	/	7 (Cauc-asians; 61 years)	Illumina HiScanSQ	DESeq2	1092 ↑, 1274 ↓; Log2 FC > 2, FDR < 0.05	/	/
[79]	Deposited to SRA: PRJNA524323	Transcript. data generation	8	4	/	/	6	Illumina HiSeq 2500	/	/	/	/
[80]	Retrieved from GEO: GSE171830	Bioinfor. data reanalysis	/	/	6	/	6	/	Limma	257 ↑, 46 ↓; log2 FC > 2; *p* < 0.01	/	/
[75]	Deposited to GEO: GSE142538	Differ. splicing and gene expression analyses	6	4	/	6 (declared as pre-sympto-matic FECD)	9	Illumina Nextseq 500/550	DSGs: rMATS (v.4.0) DEGs: Cuttdiff within Cufflinks	DEGs: 215 (CO_RE+), 1330 (F_RE+), 696 (F_RE−); FDR < 0.05	ASE: Skipped exon: 313 (CO_RE+), 1030 (F_RE+), 737 (F_RE−). Alternative 5′ splice site: 30 (CO_RE+), 63 (F_RE+), 52 (F_RE−). Alternative 3′ splice site: 7 (CO_RE+), 51 (F_RE+), 37 (F_RE−). Mutually exclusive exon: 62 (CO_RE+), 237 (F_RE+), 140 (F_RE-). Retained intron: 37 (CO_RE+), 194 (F_RE+), 184 (F_RE−); FDR < 0.001.	ASE: Confirmed in CO_RE+/F_RE+ vs. CO for MBNL2 (exon 7 inclusion), SYNE1 (exon9 exclusion), INF2 (exon 22 exclusion), MBNL1 (exon 5 inclusion), NUMA1 (exon 16 exclusion), SORBS1 (exon 23 exclusion) by RT-PCR. DEGs: Confirmed *FN1* ↑ in CO_RE+, *COL4A2* ↑ in CO_RE+, *COCH* ↑ in CO_RE+, *MSI1* ↑ in CO_RE+, *LUM* ↓ in F_RE+, *KDR* ↓ in F_RE+ and CO_RE+, *SOD3* ↓ in F_RE+ by qPCR
[81]	Deposited to GEO: GSE75676	Differ. gene expression analysis	/	/	4 (late- onset, 75.5 yeras)	/	4 (52.8 years)	Affymetrix GeneChip Human Gene 1.0 STA	Moderate *t* statistic	487 ↑, 467 ↓; Log2 FC > 2, FDR < 0.05	/	Confirmed ↑ *SLC4A11*, *COL4A5*, *COL4A6*, *FGFR2*, *SULF1*, *HLA*-*DQB1*, *BMP3*, *COL1A1*, *ITGB2*, *SERPINA1*, *FGF7*, *FOSB*, *CCR1*, *NOX4*, *BMP6*, *SELPG*, *JUN*, *PALLD*, *CD74*, *HLA-DQA1*, *BMP4*, *CSF1R*, *CD86*, *HLA-DMB*, *ITGB8*, *EDN3*, *EDN1*, *CD4*, *FGF9*, *BDNF*, *HLA-DPA1*, *TNFRSF11B*, *FN1*, *CX3CR1*, *IGF1*, *LYVE1*, and ↓ *TAC1*, *SERPINA3*, *MMP10*, *SOD2*, *CXCL1*, *ICAM1*, *TLR2*, *SOD3*, *PLAUR*, *TGFBI*, *NFKBIA*, *MMP14*, *TIMP1* by qPCR. Confirmed ↑ *HLA*-*DRA* by qPCR/IHC. Confirmed ↑ ACTA2, KRT7, SOD3 and ↓ SERPINA3 by IHC [82]
[83]	Extracted from GEO: GSE74123	Bioinfor. data reanalysis	/	/	4 (late-onset, 75.5 years)	/	4 (52.8 years)	/	Linear correlation	592 ↑, 527 ↓; Log2 FC > 1; *p* < 0.05	/	/
[84]	Retrieved from GEO: GSE10187	Differ. gene expression	/	/	5	/	2	/	ARCHS4 gene expression matrix v5	50 DEGs	/	/
[85]	Retrieved from own study [78] and from SRA: DRP006678, DRA015078	Bioinfor. data reanalysis	No data	No data	No data	No data	No data	/	DESeq2 package (v1.34.0)	/	1 (TCF4-277 isoform of *TCF4*); Log2 FC ≥ 1.5; *p* < 0.05	/
[86]	Extracted from GEO: GSE112201	Differ. gene expression	/	/	10	/	3	Illumina NovaSeq 6000	DESeq2	lncRNA *NEAT1* ↓; *p* < 0.01, log2 FC > 1.5	/	Confirmed by qPCR

### 4.2. Altered Gene-Expression Patterns

As an addition to differential splicing events’ analysis, Chu et al. [75] also applied differential gene-expression analysis between study groups. In comparison to the control group, they identified 1330 DEGs in the FECD group with intronic TNR expansion in *TCF4*, 696 DEGs in the FECD group without intronic TNR expansion in *TCF4* (602 DEGs shared with the FECD group with intronic TNR expansion), and 215 DEGs in the pre-symptomatic group (73 DEGs shared with the FECD group with intronic TNR expansion). Using quantitative PCR (qPCR), they confirmed different gene-expression patterns of *FN1*, *COL4A2*, *COCH*, *MSI1* and *KDR* in the pre-symptomatic group compared to the control group and downregulation of *LUM*, *KDR* and *SOD3* in the FECD group with intronic TNR expansion compared to the control group. Due to the similarity of alternative splicing patterns between the non-symptomatic and the FECD group with intronic TNR expansion in *TCF4*, the authors hypothesized that changes in gene expression in endothelial cells with pathologic intronic TNR expansion in *TCF4* are present long before the beginning of FECD symptoms, which could be an important resource to identify candidate biomarkers for earlier diagnosis and treatment of FECD [75].

Kuot et al. [76] identified 142 DEGs when applying a microarray gene expression of corneal endothelium between pooled RNA samples of FECD cases and individual RNA controls. Provided DEGs were subjected to enrichment analysis using the InnateDB tool, and processes related to ECM reorganization, cellular remodeling, immune response, and inflammation were retrieved. The upregulated expression of *ALPK2*, *BGN*, *CLIC6*, *CST1*, *CX3CR1*, *EDN1*, *HLA-DRA*, *NOX4* and the downregulated expression of *CPAMD8* and *PPP1R1B* in FECD cases were confirmed by RT-qPCR.

Nakagawa et al. [78] identified 2366 DEGs (1092 up- and 1274 downregulated) when performed RNA-seq comparing late-onset FECD cases (n = 10) to healthy controls (n = 7). The identified DEGs were subjected to a series of enrichment analyses using the Gene Ontology (GO) database for biological process, cellular components, and molecular function, and the Reactome and Kyoto Encyclopaedia of Genes and Genomes (KEGG) databases for biological pathways. The upregulated genes were enriched in GO biological processes of extracellular structure organization, in GO cellular components of collagen-containing ECM, endoplasmic reticulum lumen, and secretory granule membrane, and in the GO molecular function of ECM structural constituents, glycosaminoglycan binding, and peptidase regulator activity. Enriched KEGG/Reactome pathways of upregulated genes were associated with phosphatidylinositol 3-kinase (PI3K)/Akt signaling pathway, focal adhesion, ECM organization, signaling by receptor tyrosine kinase, and degradation of the ECM. The downregulated genes were enriched in GO biological processes of response to oxidative stress, regulation of the apoptotic signaling pathway and epidermis development, in GO cellular components of cell–cell junctions, nuclear specks and cell–substrate junctions, and in the GO molecular function of nucleoside binding, ribonucleoside binding, and purine ribonucleoside binding. Enriched KEGG/Reactome pathways of downregulated genes were associated with mitogen-activated protein kinase (MAPK) signaling pathways, apoptosis, the p53 signaling pathway, the NF-kappa B signaling pathway, cellular response to external stimuli, cellular response to stress, signaling by interleukins, programmed cell death and cellular senescence. According to the enriched terms and pathways, an excessive production of ECM was suggested to be an important factor in FECD pathophysiology [78].

### 4.3. Reanalysis of Transcriptomics Data

High-throughput platforms generate enormous datasets that can be stored on repositories such as Gene-Expression Omnibus (GEO) or NCBI Sequence Read Archive (SRA) to become publicly accessible to other research groups. Datasets of various studies can be combined to increase sample size and statistical power in a diverse range of downstream bioinformatics analyses [87].

In this way, Zhang et al. [71] performed a transcriptomic meta-analysis of corneal endothelium in FECD patients with and without intronic TNR expansion in *TCF4*. They combined their own RNA-seq dataset (GEO, GSE142538) from a previous study [75] with additional RNA-seq datasets extracted from SRA (PRJNA524323), provided by Nikitina et al. [79], and from GEO (GSE112201), provided by Wieben et al. [74]. Altogether, they obtained transcriptomics data of 25 FECD cases with intronic TNR expansion in *TCF4*, 8 FECD cases without intronic TNR expansion in *TCF4*, and 19 controls (without intronic TNR expansion in *TCF4*), which were further used for differential expression and alternative splicing analyses. A large difference in the numbers of obtained DEGs and DSGs between compared groups was observed when they separately analyzed the transcriptomics datasets. After transcriptomics data synthesis, similar transcriptomic signatures were observed between FECD patients with or without intronic TNR expansion in *TCF4*. However, they still identified 1184 significantly up- and 1018 significantly downregulated genes in both patient groups when separately compared to the control group. Identified genes were annotated in significant biological pathways included in cell signaling, ECM organization, RNA splicing, mitochondrial, and energy metabolism (the last two pathways were the most highlighted) [71]. In another meta analysis [77] of five transcriptomics studies, a great variation in DEGs was also observed when analyzing each study separately. However, 1537 genes were found to be consistently dysregulated in the FECD cohort, including the newly identified *MCOLN3*, *MFNG*, and *ITGAM*. Further functional and pathways analysis of top 100 down- and upregulated genes revealed enriched immune-related signaling pathways.

Cui et al. [83] reanalyzed a microarray dataset (GSE74123) obtained from corneal endothelial of late-onset FECD cases and normal controls and identified significantly 592 up- and 527 down-expressed genes in FECD group. The upregulated genes were enriched in GO terms associated with immune response, ECM organization, the positive regulation of cell proliferation and leukocyte migration, while downregulated genes were enriched in the oxidation–reduction process, the negative regulation of cell proliferation/apoptotic processes, and visual perception. A network of pathways involving cell senescence, EMT, immune response, and ECM was suggested as possible pathological molecular mechanisms of late-onset FECD [83].

Honda et al. [85] utilized their own RNA-seq dataset and additional transcriptomic datasets (PRJNA524323 and PRJNA597342) of corneal endothelium from FECD patients with and without intronic TNR expansion in *TCF4* and normal controls to identify the TCF4 isoforms characterizing FECD. First, they analyzed only their own transcriptomics data, and three up- (named TCF4-283, TCF4-218 and TCF4-255) and two downregulated (TCF4-276 and TCF4-290) TCF4 isoforms were detected in FECD cases without intronic TNR expansion compared to controls. Upregulated TCF4-277 and downregulated TCF4-232 isoforms were identified when comparing FECD cases with intronic TNR expansion and controls. When they added additional transcriptomics datasets, they observed a low overlap between study cohorts, except the TCF4-277 isoform, which was upregulated in all three datasets when comparing FECD cases with intronic TNR expansion in *TCF4* and controls. It was concluded that the TCF4-277 isoform is candidate pathogenic isoform, characterizing FECD patients with intronic TNR expansion in *TCF4* [85].

In order to explore potential new therapeutic molecular targets for corneal endothelium in FECD, Liu et al. [80] used the limma software (3.20) to reanalyze the microarray transcriptomics data from GEO (GSE171830) performed on FECD patients (n = 6) and normal controls (n = 6). Altogether, 303 DEGs (257 up- and 46 downregulated) were identified and enriched with the highest degree in pathways related to ECM organization. The identified DEGs were further constructed into a protein–protein interaction (PPI) network using String database and visualized by Cytoscape software (version 3.6.1). In that way, seven hub genes *ANXA1*, *VCAN*, *GPC3*, *TNC*, *IGFBP7*, *MATN3*, and *SPARCL1* were suggested as a key genes that may served as potential therapeutic targets for FECD [80]. Wen et al. [84] used a RNA-seq dataset (GSE101872) of corneal endothelial from FECD cases and controls and tested Library of Integrated Network-based Cellular Signatures (LINCS) prediction software 2022 to identify novel drugs that can reverse the pathological gene-expression signature. According to the retrieved enriched pathways associated with the immune response, histone deacetylase (HDAC) inhibitors such as immunomodulators trichostatin A and vorinostat were suggested as candidates to regulate cytokine production in FECD [84].

### 4.4. Non-Coding RNA

The transcriptome is composed not only of protein-coding RNAs (mRNAs), but also of non-coding RNAs (ncRNAs). Small, non-coding 20 to 24 nucleotide-long RNAs called micro RNAs (miRNAs) post-transcriptionally regulate gene expression through miRNA–mRNA base pairing. The interaction between miRNA and mRNA leads to mRNA destabilization and cleavage or direct translational repression [88]. It was shown that miRNAs play a role in FECD pathophysiology [89]. Matthaei et al. [90] applied RT-qPCR to analyze the expression of 754 miRNAs in corneal endothelium samples between age-matched FECD cases (n = 6) and normal controls (n = 6). They observed detectable levels of 311 miRNAs in tissue samples, of which 87 was significantly downregulated in FECD group, including the miR-29 family which played a role in the regulation of ECM. Validation analysis confirmed the downregulation of miR-29a-3p, miR-29b-2-5p, and miR-29c-5p miRNA transcripts and increased mRNA and protein levels of their corresponding targets collagen I, collagen IV, and laminin. They concluded that miR-29 depletion in corneal endothelium may lead to the accumulation of the ECM and thickening of the Descemet membrane in FECD patients [90].

According to our knowledge, no transcriptomics study has been performed on small non-coding RNAs (sncRNAs) or long non-coding RNAs (lncRNA) in FECD.

### 4.5. Single-Cell RNA-Seq

Wang et al. [86] applied single-cell RNA-seq on four healthy corneas to construct human corneal endothelium atlas. It was demonstrated that the corneal endothelium is heterogeneous, exhibiting four subpopulations of cells named C0, C1, C2, and C3 with unique gene-expression patterns. The GSEA of each endothelial cell population was assessed to gain an insight into their biological role. On the basis of the identified enriched pathways associated with ion transport, glycolysis, response to oxygen and ECM organization, the authors hypothesized that C0 cells play a core role in corneal endothelial function to maintain endothelial structure and pump fluid.

To identify the molecular discrepancy in corneal endothelium in FECD, the authors further obtained bulk RNA-seq data (GEO, GSE112201) and compared expression patterns of 21 FECD-associated genes with the transcriptomics data of normal endothelial cell subpopulations. They found that most of FECD-associated genes were enriched in C0 cells, including *TCF4*, *TGFB1*, and *COL8A2*. When they checked for the highest gene-expression levels in C0 cells, they observed the nuclear-enriched abundant transcript 1 (*NEAT1*) as the most characteristic lncRNA. Interestingly, *NEAT1* was shown to be significantly downregulated in FECD cases when analyzing FECD bulk RNA-seq data. In a functional study using a mouse model, it was demonstrated that suppression of *Neat1* and UVA exposure reproduced the phenotype of late-onset FECD. On the other hand, the overexpression of *Neat1* protected the cornea from UVA-induced FECD. It was concluded that C0 cells act as effector cells and are more vulnerable to genetic perturbations and factor risk for FECD [86].

During our literature search, we have not found any study performing single-cell RNA-seq on corneal endothelial cells from FECD patients.

## 5. Epigenomics of FECD

During the literature search, we have found three studies analyzing global the DNA methylation of corneal endothelium in FECD patients, which are summarized in Table 3.

Khuc et al. [91] compared the DNA methylom between normal (n = 4) and FECD (n = 9) corneal endothelial samples using a DNA methylation array. Out of a total 482421 probes, 100,961 differentially methylated probes were identified; from which 6439 were hyper- and 4531 were hypo-methylated in FECD group. Most of the differentially methylated probes were annotated to the intergenic regions, especially in promoter regions. The authors highlighted top 20 differentially methylated probes that targeted protein-coding genes, including *PDE11A*, *CCDC57*, *GNAS*, *MTUS2*, *COBL*, *SPG21*, *NME6*, *CDH4*, *MYADML*, *GUCY2C*, *BSN*, and *CCDC124*, which were mainly enriched in pathways associated with cytoskeletal organization [91]. In the latter analysis [92], it was revealed that the majority of differentially methylated patterns in FECD are associated with the promoter hypermethylation of miRNA genes. Among others, *miR-199B* was found to be hypermethylated, which is a negative regulator of the expression of two zinc-finger transcription factors, *SNAI1* and *ZEB1*, which play a role in ECM production. The promoter hypermethylation of five identified miRNA genes in a FECD cohort, *miR*-*199A1*, *miR-874*, *miR-140*, *miR-23B*, and *miR-1306*, was confirmed by RT-PCR [92].

Westin et al. [93] analyzed DNA methylation patterns between FECD patients with intronic TNR expansion in *TCF4* (n = 17) and normal controls without intronic TNR expansion in *TCF4* (n = 11). They observed age-associated hypomethylation in elderly controls (≥57 years) compared to younger controls (<30 years). Therefore, case–control comparisons were made using only age-matched controls. This way, 3488 CpG sites with different DNA methylation patterns were identified; of which 1983 CpGs were hypo- and 1505 CpGs were hyper-methylated in FECD. The most hyper- and hypo-methylated regions were located in the predicted promoters of the aquaporin 1 (*AQP1*) and the coagulation factor V (*F5*), respectively. The authors also assessed the impact of altered methylation status to the gene-expression levels. They detected variable *AQP1* expression levels among FECD cases, while *F5* expression was significantly increased in FECD patients compared to controls. On the other hand, protein F5 level was similar in both study groups. No difference in DNA methylation patterns of any known candidate genes associated with FECD (*COL8A2*, *ZEB1*, *SLC4A11*, *LAMC1*, *LOXHD1*, *KANK4*, *ATP1B1* and *DMPK*) was observed. The authors concluded that intronic TNR expansion of *TCF4* has no impact on the global DNA methylation in patients with FECD [93].

**Table 3:** Extracted data from epigenomics studies performed on corneal endothelium in FECD patients. F_RE+ refers to FECD patients with confirmed intronic trinucleotide repeat (TNR) expansion in *TCF4*. ↑ and ↓ refer to hyper- or hypo-methylated microarray probes or CpG islands in FECD patients compared to controls. N.s. refers to FECD patients without intronic TNR expansion in *TCF4* or these data are not provided in the study. Abbreviations: FDR = false discovery rate. qPCR = quantitative polymerase chain reaction. GEO = Gene-Expression Omnibus database. / indicates no data/information in the original study.

Ref.	Raw Data: Accession Number	Study Design	Fuchs Group (Average Age)		Control Group (Average Age)	Genome-Wide Platform	Bioinformatics Tools/Program	Loci Associated with FECD (Hypo (↓) or Hyper (↑) Methylated)	Validation
			F_RE+	n.s.					
[91]	Deposited to GEO: GSE94462	Diff. methyated genes	/	9 (64 years)	4 (71 years)	Illumina Infinium HumanMethylation450	Probe-wise linear model	6439 probes ↑, 4531 probes ↓; FDR < 0.05	Confirmed *MIR199B* ↑
[92]	Extracted from GEO: GSE94462	Diffe. methylated miRNAs	/	9	4	/	Sample pairwise correlation in R program	154 probes ↑, 62 probes ↓; FDR < 0.01	Confirmed *miR-199A1* and *miR-23B* ↑ by qPCR
[93]	Deposited to GEO: GSE198917	Differ. methylated CpG islands	16	/	9	Illumina Human Infinium MethylationEPIC array	Minfi package (v. 1.32)	1505 CpGs ↑, 1983 CpGs ↓; FDR < 0.05	/

## 6. Proteomics of FECD

The altered expression of *TCF4* with intronic TNR expansion was also confirmed at the protein level in the corneal endothelium of FECD patients [94]. As previously identified by transcriptomic analysis [81], De Roo et al. [82] used the immunohistochemical (IHC) staining of paraffin-embedded corneal endothelial cells and confirmed a higher expression of alpha-smooth muscle actin (ACTA2), cytokeratin 7 (KRT7), superoxide dismutase 3 (SOD3) and the decreased expression of serpin peptidase inhibitor clade A member 3 (SERPINA3) in FECD compared to control group. It was hypothesized that EMT and oxidative stress play a role in FECD [82]. During the literature search, we obtained three proteomics studies, which are summarized in Table 4.

Since aqueous humor surrounds the corneal endothelium, it has been used in proteomics analyses to identify potential correlations with dysfunctional endothelium in FECD. Richardson et al. [95] applied liquid chromatography–tandem mass spectrometry (LC-MS/MS) analyses to compare aqueous humor proteomes between patients with (n = 12) and without (n = 11) FECD. They identified 64 proteins in the albumin-depleted fraction and 34 proteins in the albumin-bound fraction. In the fraction with 64 isolated proteins, afamin (AFM), complement C3 (C3), histidine-rich glycoprotein (HRG), immunoglobulin heavy (IgH) and protein family with sequence similarity 3, member C (FAM3C) were significantly up-, and suprabasin (SBSN) was significantly downregulated in FECD group. In the fraction with 34 proteins, hemoglobin fragment, immunoglobulin kappa (IgK), Ig lambda (IgL) and uncharacterized protein albumin were significantly upregulated in FECD group [95].

FECD is characterized by the appearance of pathological guttae and the thickening of Descemet’s membrane. Kuot et al. [96] applied nanoscale ultra-performance liquid chromatography–mass spectrometry (nUPLC-MS) on Descemet’s membrane samples to identify proteomics changes between FECD cases (n = 3) and controls (n = 3). A total of 55 proteins were detected, of which 18 were present in both groups, 15 only in the FECD cases, and 22 only in the controls. The significant downregulation of apolipoprotein E (APOE) and immunoglobulin-heavy constant gamma 1 protein (IGHG1) was observed in Descemet’s membrane of the FECD group. Downregulation of the *APOE* gene and protein was confirmed in FECD corneal endothelium by RT-qPCR and IHC, respectively, while the gene-expression levels of *IGHG1* was too low for conclusions [96]. Nakagawa et al. [97] conducted shotgun proteomics of the Descemet’s membrane, including guttae, to provide dysregulated proteins in FECD. They identified 32 proteins, including biglycan (BGN), collagen-type VI alpha 2 chain (COL6A2), collagen-type VIII alpha 1 chain (COL8A1), collagen-type XVIII alpha 1 chain (COL18A1), latent transforming growth factor beta-binding protein 2 (LTBP2), lumican (LUM), matrilin 2 (MATN2), matrilin 3 (MATN3), mucin 6 oligomeric mucus/gel-forming (MUC6), proline/arginine-rich end leucine-rich repeat protein (PRELP) and tenascin C (TNC), which were only expressed in FECD patient when compared with Descemet’s membrane of the normal control. The GO analysis of differentially expressed proteins revealed pathways associated with ECM. The exclusive expression of the top 4 of the 32 proteins (hemoglobin α (HBA1), Sushi repeat-containing protein (SRPX2), TNC and hemoglobin γδεβ), and the overexpression of fibrinogen α (FGA) in FECD cases was also confirmed by IHC technique [97].

**Table 4:** Extracted data from proteomics and metabolomics studies performed on FECD. Abbreviations: qPCR refers to quantitative polymerase chain reaction. IHC = immunohistochemistry. / indicates no data/information in the original study.

PROTEOMICS of FECD								
Ref.	Tissue Source	Raw Data: Accession Number	FECD Group (Ancestry, Average Age)	Control Group (Average Age)	Genome-Wide Platform	Bioinformatics Tools/Program	Differentially (↑ = Up- and ↓ = Downregulated) Expressed Proteins/Metabolites Associated with FECD Group	Validation of Identified Loci
[95]	Aqueous humor	/	12 (coexisted cataracts, 62.8 years)	11 (with cataract, 64.0 years)	Liquid chromatography–tandem mass spectrometry	Student’s t-test	↑: SBSN, hemoglobin fragment (n.s.), immunoglobulin kappa (n.s.), immunoglobulin lambda (n.s.), uncharacterized protein albumin (n.s.), ↓: AFM, C3, HRG, IGH (n.s.), FAM3C; *p* ≤ 0.01	/
[96]	Descemet’s membrane	/	3 (Caucasians, 72.3 years)	3 (Caucasians, 85.3 years)	Nanoscale ultra-performance liquid chromatography–mass spectrometry	PLGS Expression Analysis Software (v.2.4.) Two-tailed Student *t*-test	APOE and IGHG1 ↓; *p* < 0.05	Confirmed *APOE* ↓ gene (in CE) and protein expression by qPCR and IHC
[97]	Descemet’s membrane	Deposited to MassIVE: MSV000091078	1 (63 years old)	1 (80 years old)	Liquid chromatography–tandem mass spectrometry	n.s.	32 exclusive proteins	Confirmed exclusive expression of HBA1, SRPX2, TNC, hemoglobin γδεβ) (n.s.) and ↑ of FGA by immunofluorescence staining
METABOLOMICS of FECD								
[98]	Aqueous humor	/	8 (56.8 years)	10 (50.8 years)	Lipidomic ultra-performance liquid chromatography–mass spectrometry	Univariate analysis; Student’s *t*-test.	23 ↑, 4 ↓ lipids; *p* < 0.05	/

## 7. Metabolomics of FECD

An early study using chromatographic testing demonstrated altered concentrations of the free amino acids threonine, asparagine, glutamine, phosphoserine, phosphoethanolamine, and arginine in the aqueous humor of patients with FECD [99].

During the literature search, we found one metabolomics study (Table 4). Cabrerizo et al. [98] applied ultra-performance liquid chromatography–mass spectrometry to detect lipidomic differences in aqueous humor in patients with FECD compared with controls. They reported 27 lipids with significantly changed levels, of which 24 showed increased levels. The majority of these lipids belonged to the chemical groups of diacylglycerophosphocholines, 1-ether, 2-acylglycerophosphocholines, sphingomyelins, and cholesteryl esters. It was hypothesized that lipid overexpressed in the aqueous humor of FECD patients may reflect increased intracellular oxidative stress due to the unfolded protein response in corresponding corneal endothelium [98].

## 8. Data Synthesis

We performed a data synthesis of highlighted loci associated with FECD at particular omics levels in original studies to perform GSEA for candidate biological terms and pathways (Table 5).

When we sorted the retrieved studies according to genomics (Table 1), transcriptomics (Table 2), epigenomics (Table 3), or proteomics (Table 4) level, we extracted the highlighted loci that were identified to be associated with FECD. In many original studies, FECD groups were formed based on the results of DNA genotyping for the presence or absence of intronic TNR expansion in *TCF4*. Therefore, we established gene lists by performing data synthesis based on comparisons of FECD groups according to *TCF4* genotyping results. Gene lists were uploaded to the Database for Annotation, Visualization, and Integrated Discovery (version DAVID 2021) [100] for functional annotation to obtain enriched biological terms, including GO biological process, GO cellular component, GO molecular function, and KEGG and Reactome pathways. Enriched term/pathways were significant when false discovery rate (FDR) < 0.05.

Most of the significantly enriched pathways were identified when we analyzed gene lists that were constructed from original studies using FECD cases without information regarding *TCF4* genotyping. Most of the enriched GO terms and pathways in all omics layers were associated with ECM, which is in accordance with the literature.

**Table 5:** Results of gene-set enrichment analyses (GSEA) using synthesized data from omics studies performed on FECD. Original studies were first sorted according to omics level, and highlighted loci identified between FECD case and control groups comparison were extracted. F_n.s. refers to an FECD group where genotyping information for intronic TNR expansion in *TCF4* was not reported. F_RE+ refers to an FECD group with a confirmed presence of intronic TNR expansion in *TCF4*. F_RE− refers to an FECD group with a confirmed absence of intronic TNR expansion in *TCF4*. CO_RE+ refers to pre-symptomatic corneas from deceased donors with confirmed intronic TNR expansion in *TCF4*. Abbreviations: GO = Gene Ontology. CC = cellular component. MF = molecular function. BP = biological process. KEGG = Kyoto Encyclopedia of Genes and Genomes. / indicates no enriched term or pathway.

Synthesized Highlighted Loci That Were Identified by Two-Group Comparison in Original Studies	Gene Ontology Database	GO_TERM/KEGG/Reactome Pathway	Annotated Genes	FDR Value	Omics Level
**Comparison of GSEA results between F_n.s. vs. CO groups**					
*TCF4*, *KANK4*, *LAMC1*, *RORA*, *LAMA5*, *LINC00970*, *ATP1B1*, *LAMB1*, *PIDD1*, *COL18A1*, *SSBP3*, *THSD7A*, *HS3ST3B1*, *ND3*	GO_TERM_CC	GO:0043259~laminin-10 complex	*LAMA5*, *LAMB1*, *LAMC1*	**3.17 × 10^−5^**	Genomics
	GO_TERM_BP	GO:0007155~cell adhesion	*LAMA5*, *COL18A1*, *LAMB1*, *LAMC1*, *ATP1B1*	**0.0232**	
	GO_TERM_MF	GO:0005201~extracellular-matrix structural constituent	*LAMA5*, *LAMB1*, *LAMC1*	0.0903	
	KEGG_PATHWAY	hsa04512: ECM-receptor interaction	*LAMA5*, *LAMB1*, *LAMC1*	**0.0287**	
	REACTOME_PATHWAY	R-HSA-3000157~Laminin interactions	*LAMA5*, *COL18A1*, *LAMB1*, *LAMC1*	**1.04 × 10^−4^**	
*PDE11A*, *CCDC57*, *GNAS*, *MTUS2*, *COBL*, *SPG21*, *NME6*, *CDH4*, *MYADML*, *GUCY2C*, *BSN*, *CCDC124*	GO_TERM_CC	GO:0005813~centrosome	*CCDC57*, *CCDC124*, *MTUS2*	1	Epigenomics
	GO_TERM_BP	/	/	/	
	GO_TERM_MF	/	/	/	
	KEGG_PATHWAY	hsa00230: Purine metabolism	*GUCY2C*, *PDE11A*, *NME6*	0.1186	
	REACTOME_PATHWAY	R-HSA-418346~Platelet homeostasis	*PDE11A*, *GNAS*	1	
*ALPK2*, *BGN*, *CLIC6*, *CST1*, *GPC3*, *CX3CR1M*, *EDN1*, *HLA-DRA*, *NOX4*, *CPAMD8*, *PPP1R1B*, *ANXA1*, *VCAN*, *TNC*, *IGFBP7*, *MATN3*, *SPARCL1*	GO_TERM_CC	GO:0062023~collagen-containing extracellular matrix	*VCAN*, *ANXA1*, *BGN*, *TNC*, *GPC3*, *SPARCL1*, *IGFBP7*, *MATN3*	**3.11 × 10^−7^**	Transcriptomics
	GO_TERM_BP	GO:0071385~cellular response to glucocorticoid stimulus	*EDN1*, *ANXA1*	1	
	GO_TERM_MF	GO:0005201~extracellular-matrix structural constituent	*BGN*, *TNC*, *IGFBP7*, *MATN3*	**0.0082**	
	KEGG_PATHWAY	hsa04933: AGE-RAGE signaling pathway in diabetic complications	*EDN1*, *NOX4*	1	
	REACTOME_PATHWAY	R-HSA-8957275~Post-translational protein phosphorylation	*VCAN*, *TNC*, *GPC3*, *SPARCL1*, *IGFBP7*, *MATN3*	**2.57 × 10^−6^**	
*ACTA2*, *KRT7*, *SOD3*, *SERPINA3*, *AFM*, *C3*, *HRG*, *FAM3C*, *SBSN*, *APOE*, *HBA1*, *SRPX2*, *TNC*, *COL6A2*, *COL8A1*, *COL18A1*, *LTBP2*, *LUM*, *MATN2*, *MATN*, *MUC6*, *PRELP*, *TNC*, *FGA*	GO_TERM_CC	GO:0062023~collagen-containing extracellular matrix	*FGA*, *COL18A1*, *SERPINA3*, *LUM*, *TNC*, *PRELP*, *LTBP2*, *SOD3*, *SRPX2*, *COL6A2*, *COL8A1*, *APOE*, *HRG*, *MATN2*	**3.67 × 10^−16^**	Proteomics
	GO_TERM_BP	GO:0001525~angiogenesis	*COL18A1*, *SRPX2*, *COL8A1*, *HRG*	0.6518	
	GO_TERM_MF	GO:0005201~extracellular matrix structural constituent	*FGA*, *SRPX2*, *LUM*, *TNC*, *LTBP2*, *PRELP*, *MATN2*, *MUC6*	**1.95 × 10** **^−9^**	
	KEGG_PATHWAY	hsa04974: Protein digestion and absorption	*COL18A1*, *COL6A2*, *COL8A1*	0.1611	
	REACTOME_PATHWAY	R-HSA-216083~Integrin cell surface interactions	*FGA*, *COL18A1*, *LUM*, *COL6A2*, *TNC*, *COL8A1*	**2.68 × 10** **^−5^**	
**Comparison of GSEA results between F_RE+ vs. CO groups**					
*TCF4*, *MBNL1*, *INF2*, *ITGA6*, *ADD3*, *SORBS1*, *NUMA1*, *KDR*, *PPFIBP1*, *MBNL2, INF2, SOD3*, *SORBS1*, *SYNE1*, *MBNLL1*, *MBN2*, *COCH*, *LUM*	GO_TERM_CC	GO:0005938~cell cortex	*PPFIBP1*, *NUMA1*, *ADD3*	0.4548	Transcriptomics
	GO_TERM_BP	GO:0031589~cell–substrate adhesion	*ITGA6*, *SORBS1*	1	
	GO_TERM_MF	GO:0045296~cadherin binding	*PPFIBP1*, *KDR*, *ITGA6*	0.8334	
	KEGG_PATHWAY	hsa04820: Cytoskeleton in muscle cells	*INF2*, *ITGA6*, *SYNE1*	0.1618	
	REACTOME_PATHWAY	R-HSA-216083~Integrin cell surface interactions	*LUM*, *KDR*, *ITGA6*	0.1557	
**Comparison of GSEA results between F_RE+ vs. F_RE− groups**					
*MBNL1*, *NUMA1*, *APBB2*, *PPFIBP1*, *INF2*, *SCARB1*, *SYNE1*, *ADD3*, *MBNL2*, *TTC7A*, *ARVCF*, *TSPOAP1*, *NDUFV3*, *IFI44*, *EXOC1*, *ITGA6*, *CLASP1*, *COPZ2*, *CD46*, *CADM1*	GO_TERM_CC	GO:0005938~cell cortex	*PPFIBP1*, *NUMA1*, *ADD3*, *CLASP1*	0.0516	Transcriptomics
	GO_TERM_BP	GO:0007010~cytoskeleton organization	*ADD3*, *CLASP1*, *SYNE1*	0.9175	
	GO_TERM_MF	GO:0045296~cadherin binding	*PPFIBP1*, *ARVCF*, *ITGA6*, *CD46*	0.1911	
	KEGG_PATHWAY	hsa04820: Cytoskeleton in muscle cells	*INF2*, *ITGA6*, *SYNE1*	0.4008	
	REACTOME_PATHWAY	R-HSA-380320~Recruitment of NuMA to mitotic centrosomes	*NUMA1*, *CLASP1*	1	
**Comparison of GSEA results between CO_RE+ vs. CO groups**					
*INF2*, *NUMA1*, *SORBS1*, *SYNE1*, *MBNL1*, *MBN2*, *KDR*, *FN1*, *COL4A2*, *COCH*, *MSI1*	GOTERM_CC_DIRECT	GO:0062023~collagen-containing extracellular matrix	*COL4A2*, *FN1*, *COCH*	0.9182	Transcriptomics
	GOTERM_BP_DIRECT	GO:0008360~regulation of cell shape	*KDR*, *FN1*, *COCH*	0.2912	
	GOTERM_MF_DIRECT	GO:0003779~actin binding	*INF2*, *SORBS1*, *SYNE1*	0.6847	
	KEGG_PATHWAY	hsa04820: Cytoskeleton in muscle cells	*INF2*, *COL4A2*, *FN1*, *SYNE1*	**0.0073**	
	REACTOME_PATHWAY	R-HSA-216083~Integrin cell surface interactions	*COL4A2*, *KDR*, *FN1*	0.0802	

## 9. Discussion

FECD is thought to be a complex multifactorial and progressive disease involving genetic and environmental risk factors that cause degeneration of the cornea endothelium and Descemet’s membrane. In this review, we gathered genome-wide studies (n = 31) of different omics levels which have either provided new or reanalyzed high-throughput data to determine the molecular background of FECD (Table 1, Table 2, Table 3 and Table 4).

At the genomics level (Table 1), the *TCF4* gene demonstrated the strongest association with FECD. At the transcriptomics level (Table 2), gene expression and splicing patterns in the corneal endothelium of FECD patients with the presence or absence of pathogenic intronic TNR extension in *TCF4* were studied to a great extent. The expression and accumulation of intronic TNR extension-containing TCF4 (poly(CUG)*n* TCF4) transcripts in RNA foci have been associated with the pathophysiology of FECD. Additional genes with altered splicing and expression patterns have been identified by genome-wide platforms when comparing FECD patients with or without pathogenic intronic TNR extension in *TCF4*. At the epigenomics level (Table 3), it currently appears that the presence of intronic TNR expansion in *TCF4* has no significant effect on the DNA methylome in the corneal endothelium of FECD patients, but its changes are age-related. Nevertheless, increased levels of the promoter hypermethylation of miRNA genes associated with ECM regulation, a major molecular pathomechanism of FECD, have been detected. It could be that the dysregulation of sncRNA transcripts plays an important role in the corneal endothelium of patients with FECD; however, no transcriptomic study conducted on sncRNA or lncRNA was found during our literature search. At the proteomics level (Table 4), exclusive or altered protein-expression levels in the aqueous humor or Descement’s membrane have been observed in FECD patients compared to controls. In addition, it was shown by a metabolomics study (Table 4) that the aqueous humor of FECD patients also exhibits an altered lipidomic profile.

After sorting 31 retrieved genome-wide studies according to the performed omics level, we extracted the highlighted loci associated with FECD to perform the data synthesis for GSEA. We combined loci into gene lists according to comparisons of similar study cohorts in original studies, which were established on a basis of the results of participant genotyping for the presence or absence of intronic TNR expansion in *TCF4*. Constructed gene lists of relevant omics levels were applied for enrichment analyses and pathways associated with the extracellular matrix were identified as the most prominent (Table 5). Obtained pathways are in general accordance with the literature; therefore, they may serve as a source for candidate gene selection for further studies on FECD.

The genome-wide approach provides a huge resource of candidate genes/proteins that can be further studied in cell cultures and/or animal models to determine their biological role in the investigated disease. In this way, Wang et al. [86] demonstrated that lncRNA NEAT1 has a role in protecting the corneal endothelium against oxidative damage. First, they applied RNA-seq analyses, where the high expression level of NEAT1 in healthy endothelial cells, but its significant downregulation in FECD endothelium, was observed. Further, they conducted functional studies using cell-line and mouse models to investigate the potential regulatory role of the candidate *NEAT1* in FECD pathogenesis. Increased cellular ROS levels and significantly decreased cell viability were observed in a human corneal endothelium cell line with the induced *NEAT1* knocked down when treated with H_2_O_2_ compared to non-treated cells. The potential antioxidant role of *NEAT1* was also confirmed when used a non-genetic FECD mouse model whose corneal exposure to UVA irradiation induces the symptoms of late-onset FECD. Namely, the knockdown of *Neat1* expression in corneal endothelium prior to UVA radiation exposure resulted in a reduced endothelial cell density and hexagonal morphology compared to normal control mice. Conversely, an induced overexpression of *Neat1* protected mice from the progression of FECD against UVA exposure, indicating the protective role of this lncRNA, which could be a new therapeutic strategy for FECD [86].

Corneal endothelial transplantation is currently the main treatment and visual restoration option for advanced FECD [7]. However, corneal tissue shortage is a major limiting factor for the operative treatment of FECD patients, and donated corneas are in limited supply worldwide [101], triggering a constant search for alternative non-operative or minimally invasive treatment approaches in the future. Alternative treatment options using cell cultures [102], antioxidants [103], exosome complex degradation [104], and antisense oligonucleotides [105] have been tested in FECD with encouraging results, described in more detail below. Numa et al. [102] enrolled 11 patients, including 7 FECD patients, with pseudophakic endothelial failure conditions to test the safety and efficacy of cell injection therapy using cultured human corneal endothelial cells and a Rho-associated protein kinase inhibitor, which promotes corneal endothelial cell engraftment. During a five-year follow-up period, restored normal endothelial function was observed in 10 patients, characterized by increased cell density, decreased coefficient of variation in cell size, and increased cell hexagonality. In addition, no major adverse reactions directly related to the cell injection therapy were observed, concluding that it is a safe and effective minimally invasive procedure in the clinical setting [102]. Kim et al. [103] used cultured bovine corneal endothelial cells with induced oxidative and endoplasmic reticulum stress and the L450W transgenic knock-in alpha 2 collagen VIII (Col8a2) mouse model with early-onset FECD to investigate the protective role of N-acetyclcysteine, a thiol-containing antioxidant. They detected increased cell viability rate in N-acetyclcysteine pre-treated cells compared to untreated control cells, which was manifested by a significant upregulation of the antioxidant gene *iNos* and a significant downregulation of the endoplasmic reticulum stress/unfolded protein-response genes *Grp78* and *Chop*. Transgenic mice treated with N-acetyclcysteine in drinking water also exhibited higher corneal endothelial cell density, mild variations in endothelial cell size and morphology, and had few guttae compared to untreated control mice, which showed a more severe phenotype of FECD [103]. Angelbello et al. [104] obtained corneal endothelial cells from FECD patients and incubated them with an RNA-targeting small molecule, which was designed to bind to the toxic repeat-expansion region of the poly(CUG)*n* TCF4 transcript, facilitating excision and further degradation by the nuclear RNA exosome complex. After 24 h of incubation, decreased levels of poly(CUG)*n* TCF4 transcripts and eliminated RNA toxicity in treated compared to untreated cells were detected by qPCR and cell viability assay, respectively [104]. Chau et al. [105] used a mouse model to demonstrate in vivo gene knockdown in cornea. Synthetic antisense oligonucleotides were delivered into corneal endothelium by intraocular injection and, consequently, the downregulation of the targeted *MALAT1* gene was measured [105].

More omics studies on FECD are required, in particular to identify potential dysregulated non-coding RNA transcripts, proteins, and metabolites. In order to ensure better harmonization of study groups, a detailed characterization of FECD cases and controls should be provided, including genotyping results for intronic TNR expansion within *TCF4*, age, ethnicity, the clinical stage of FECD and coexistence with other eye pathologies. Sharing raw data in database repositories will help scientists to combine multiple studies, increasing sample size and statistical power to identify strong candidate genes associated with dysregulation in FECD. Downstream enrichment analyses using different biological databases with an accumulating knowledge of molecular organization and interactions will provide information on the systemic roles of identified dysregulated genes.

## 10. Conclusions

We performed a literature search on genome-wide studies focusing on blood, corneal endothelium, Descemet’s membrane, or aqueous humor samples from patients with FECD. Genome-wide analyses revealed a number of molecular discrepancies, characterized by dysregulated gene expression and splicing patterns between FECD patients and normal controls and among FECD patients with or without intronic TNR expansion in *TCF4*.

At the genomics level, the *TCF4* gene had the strongest association with FECD, while at the transcriptomics level, the expression and accumulation of intronic TNR extension-containing TCF4 transcripts in RNA foci were frequently described in FECD. At the epigenomics level, increased levels of the promoter hypermethylation of miRNA genes associated with ECM regulation were reported. Few studies have been performed at the proteomics, metabolomics, or lipidomic level, but all these studies showed differences in the aqueous humor composition of the FECD group compared to the control group. We combined loci associated with FECD cohorts from the original studies and identified enriched extracellular-matrix-related pathways as the most prominent.

Additional genome-wide studies could provide a source of FECD-specific candidate genes for downstream functional studies to understand the underlying molecular mechanisms and to identify possible new therapeutic strategies for this disease. In particular, treatment options that are non-operative alternatives to corneal transplantation would help to overcome the constant shortage of corneal tissue.

## Abbreviations

DEG	Differentially expressed gene
EMT	Epithelial to mesenchymal transition
ECM	Extracellular matrix
FECD	Fuchs’ endothelial corneal dystrophy
FDR	False discovery rate
GSEA	Gene-set enrichment analysis
GO	Gene Ontology
GEO	Gene-Expression Omnibus
GWAS	Genome-wide association study
ICH	Immunohistochemistry
KEGG	Kyoto Encyclopedia of Genes and Genomes
mRNA	messenger RNA
miRNA	micro RNA
mtDNA	mitochondrial DNA
NGS	Next-generation sequencing
PCA	Principal component analysis
PCR	Polymerase chain reaction
RNA-seq	RNA-sequencing
ROS	Reactive oxygen species
sncRNA	Small non-coding RNA
lncRNA	Long non-coding RNA
SNP	Single nucleotide polymorphism
TNR	Trinucleotide repeats
UVA	Ultraviolet A
WES	Whole-exome sequencing
WGS	Whole-genome sequencing
